# The PER2:BRCA1:POU2F1(OCT-1) ternary complex represents a multi-component scaffold model for circadian gene regulation

**DOI:** 10.1016/j.nbscr.2025.100141

**Published:** 2025-11-30

**Authors:** Elizaveta Kadukhina, Siqi Jia, Linda M. Villa, Xiao Yi, Daniel G.S. Capelluto, Jonathan S. Briganti, Anne M. Brown, Carla V. Finkielstein

**Affiliations:** a Fralin Biomedical Institute at Virginia Tech Carilion, Virginia Tech, Roanoke, VA, 24016, USA; b Department of Biological Sciences, Blacksburg, VA, 24061, USA; c Data Services, University Libraries, Virginia Tech, Blacksburg, VA, 24061, USA; d Program in Genetics, Bioinformatics, and Computational Biology, Virginia Tech, Blacksburg, VA, 24061, USA; e Department of Biochemistry, Virginia Tech, Blacksburg, VA, 24061, USA; f Protein Signaling Domains Laboratory, Department of Biological Sciences, Blacksburg, VA, 24061, USA; g Fralin Life Sciences Institute, Virginia Tech, Blacksburg, VA, 24061, USA; h Center for Soft Matter and Biological Physics, Blacksburg, VA, 24061, USA

**Keywords:** Circadian rhythms, Period 2, BRCA1, Estrogen receptor, POU2F1(OCT-1), Tumor suppressor

## Abstract

The circadian clock component PER2 coordinates daily oscillations in gene expression across multiple tissues, yet its role in assembling multi-protein regulatory complexes remains incompletely understood. Here, we report that PER2 nucleates a ternary complex with the tumor suppressor BRCA1 and the transcription factor POU2F1(OCT-1) to impose circadian control on target gene promoters. Using bacterial two-hybrid screening, we identified BRCA1 as a novel PER2-interacting protein. Biochemical mapping revealed that PER2 engages BRCA1 through multiple discrete binding interfaces: PER2 spanning residues 356–574 and 683–872 interact with both the N-terminal (1–400) and C-terminal BRCT (1670–1863) domains of BRCA1. Structural modeling predicted 361 residue contacts between PER2 and BRCA1, substantially more than the 74 contacts predicted for PER2:POU2F1(OCT-1), indicating differential affinities that enable ordered complex assembly. Sequential pull-down assays demonstrated that PER2, BRCA1, and POU domain form a stable ternary complex *in vitro*, with POU2F1(OCT-1) serving as the DNA-binding platform. Electrophoretic mobility shift assays revealed that pre-assembly of PER2 with POU domain inhibits DNA binding, while BRCA1 is essential for stabilizing PER2 recruitment to DNA-bound POU2F1(OCT-1). Using *ESR1* as a functional readout, we demonstrated that this ternary complex directly regulates promoter activity. Circadian transcriptome analysis revealed that *Esr1* exhibits robust clock-dependent oscillations that are abolished in *Per1/2* double-knockout mice, while *Pou2f1* and *Brca1* maintain constitutive expression. These findings establish PER2 as a circadian scaffold that assembles multivalent protein complexes to temporally gate transcription, providing mechanistic insight into how circadian disruption can influence target gene expression.

## Highlights

• PER2 directly interacts with BRCA1 through multiple discrete binding interfaces.
• PER2, BRCA1, and POU2F1 (OCT-1) form a ternary complex that coordinately regulates ESR1 transcription.
• An ordered assembly of POU2F1 (OCT-1), BRCA1, and PER2 is required at the ESR1 promoter for proper transcriptional regulation.
• ESR1 expression exhibits circadian oscillations dependent on functional PER2 but not on rhythmic BRCA1 or POU2F1 expression.
• BRCA1 mutations or circadian misalignment may disrupt the ternary complex, linking circadian disruption to cancer risk.

## 1 Introduction

PERIOD 2 (PER2) is a core component and negative regulator of the circadian clock molecular machinery. Its expression contributes to the oscillatory, 24-h transcriptional cycle by engaging in feedback regulatory loops that inhibit the core heterodimer activator CLOCK (Circadian Locomotor Output Cycles Kaput):BMAL1 (Brain and Muscle ARNTL-Like 1), thereby stabilizes rhythmic gene expression (Takahashi, 2017). From an evolutionary standpoint, and despite different gene sequences, the architecture of the transcriptional-translational feedback loops driving circadian rhythms are conserved among diverse taxa, underscoring its fundamental importance in biological timing systems (Patke et al., 2020; Young and Kay, 2001).

The mammalian *Period* gene family comprises three paralogs (*Per1*, *Per2*, *Per3*), which arose through gene duplication events (Kwak et al., 2024), with human PER2 displaying functional distinctions despite strong structural similarity to PER1 and PER3 [UniProt O15534 (PER1); UniProt O15055 (PER2); UniProt P56645 (PER3)]. Each PER family member differentially regulates signaling pathways controlling cell proliferation and death and all three have been implicated in tumorigenesis at different regulatory levels (Gery et al., 2006; Schmutz et al., 2010; Grimaldi et al., 2010; Im et al., 2010). The human PER2 protein is a 1255 amino acid polypeptide that is subjected to extensive post-translational modifications that regulate its function (Narasimamurthy and Virshup, 2021; Chen et al., 2021; Masuda et al., 2020; Costanzo et al., 2025). Despite its functional importance and extensive study, only discrete regions of PER2 have been structurally characterized at atomic resolution (Hennig et al., 2009; Nangle et al., 2014; Michael et al., 2023), revealing a modular architecture in which ordered domains are interspersed with extensive intrinsically disordered regions (IDRs) (PDB: 6OF7, 8D7M, 8D7N, 8D7O). These IDRs are functionally significant as they confer conformational flexibility, facilitate binding of kinases and processive phosphorylation (Narasimamurthy and Virshup, 2021). IDRs also contain motifs involved in nuclear-cytoplasmic shuttling (Vielhaber et al., 2001; Yagita et al., 2002) and enable PER2 to bind multiple partners including nuclear receptors, chromatin modifiers, and transcriptional regulators (Schmutz et al., 2010; Grimaldi et al., 2010; Yang et al., 2006; Gotoh et al., 2014, 2015; Liu et al., 2018; Duez and Staels, 2008; Miki et al., 2012), endowing PER2 with regulatory functions beyond its canonical role in circadian timekeeping.

Among non-core clock regulators, PER2 binds the estrogen receptor alpha (ERα) and modulates its stability and transcriptional activity, though evidence that this interaction exhibits circadian dynamics remains limited. Accordingly, Gery et al. established that PER2 physically interacts with ERα *in vitro*, enhancing ERα degradation through the ubiquitin-proteasome pathway and thereby attenuating estrogen signaling in breast and endometrial cancer cell lines (Gery et al., 2007). Conversely, suppression of *PER2* expression leads to ERα stabilization and increased estrogen-responsive gene expression. Intriguingly, *PER2* itself is estrogen-inducible in breast cancer cells, creating a negative feedback loop in which estrogen signaling induces its own temporal regulator (Gery et al., 2007). While these are undoubtedly important findings, the work did not establish whether the PER2:ERα interaction exhibits circadian dynamics or generates outputs that temporally link estrogen signaling to the clock (Gery et al., 2007). Importantly, other reports have shown that sumoylation of CLOCK, which is stimulated by estradiol, enhances ERα-mediated transactivation in breast cancer cells (Li et al., 2013). Thus, whereas compelling experimental evidence from *in vitro* and *in vivo* systems establishes that estrogen and ERα regulate circadian gene expression, thereby providing a mechanism through which reproductive physiology and hormone release are temporally integrated with behavioral processes (Alvord et al., 2022), the reciprocal regulation, whether and how circadian clock components modulate estrogen signaling, remains substantially less well characterized.

In this study, we demonstrate that PER2 exerts transcriptional control over *Esr1*, which encodes ERα, through interactions with the tumor suppressor Breast Cancer 1 susceptibility protein type 1 (BRCA1) and the DNA-binding domain (POU) of the ubiquitously expressed Octamer-binding transcription factor 1 (OCT-1, also known as POU2F1). Using gene complementation assays, domain mapping approaches, AlphaFold structural modeling, and biochemical validation, we define for the first time the molecular determinants governing this regulatory complex and establish its role in linking circadian and estrogen signaling pathways. Furthermore, time-course RNA-seq studies in mice reveal that *Per2* and *Esr1* oscillate in phase across steroidogenic tissues, and that this rhythmic expression requires a functional circadian clock, as *Per1*^−/−^/*Per2*^−/−^ double knockout animals lack *Esr1* oscillations. Overall, our data support a model in which PER2 directly regulates *Esr1* transcription through assembly of a BRCA1:POU2F1(OCT-1) complex at the promoter, establishing a molecular mechanism by which the circadian clock controls ERα expression and, consequently, temporal patterns of hormonal signaling. This work establishes the first direct transcriptional mechanism linking a core clock component to *Esr1* regulation, reveals an unexpected function for BRCA1 in circadian gene regulation, and provides a molecular framework for understanding how circadian disruption may contribute to hormone-dependent cancer development.

## 2 Materials and methods

### 2.1 Cell culture and transient transfections

Human breast cancer MCF-7, Chinese hamster ovary-K1 (CHO), and human embryonic kidney HEK293T cell lines were obtained from the American Type Culture Collection (ATCC). MCF-7 and HEK293T cells were maintained in high-glucose Dulbecco's modified Eagle medium (DMEM), while CHO cells were cultured in F-12K medium; all media were supplemented with 10 % fetal bovine serum (FBS) and 100 μg/mL penicillin/streptomycin. MCF-7 cultures additionally contained 0.01 mg/mL bovine insulin (Sigma-Aldrich). Cells were maintained at 37 °C in a humidified 5 % CO_2_ atmosphere.

Transient transfections were performed using Lipofectamine LTX (ThermoFisher Scientific) with Opti-MEM antibiotic-free medium (ThermoFisher Scientific) according to the manufacturer's instructions. Cells were harvested 12 h post-transfection for protein analysis and reverse transcription quantitative PCR (RT-qPCR).

### 2.2 Bacterial two-hybrid screening

Protein-protein interactions were identified using the BacterioMatch II system (Agilent Technologies) according to the manufacturer's protocol. The bait plasmid (pBT-PER2, residues 1-1255) and prey plasmids from a liver cDNA library (pTRG) were co-transformed into bacterial cells and plated on both non-selective and selective media containing 5 mM 3-amino-1,2,4-triazole (3-AT). Following 24 h incubation at 37 °C, colonies growing on selective media were identified as potential interactors. Positive clones were maintained on LB agar plates containing tetracycline and chloramphenicol (Tet/Cam). Specificity of bait-prey interactions was confirmed by activation of the *aadA* reporter gene, which confers streptomycin resistance. Candidate positive colonies were transferred to dual-selective medium containing both 3-AT and streptomycin. Co-transformed pBT-LGF2/pTRG-Gal11P served as a positive control. For negative controls, pBT-PER2 was co-transformed with either empty pTRG vector or pTRG-Gal11P. Plasmid DNA was isolated from 3-AT–resistant colonies using cultures grown in LB medium with tetracycline, and all cDNA inserts were verified by DNA sequencing.

### 2.3 Immunoprecipitation and immunoblot assays

For immunoprecipitation experiments, cells were transfected with FLAG- or myc-tagged expression constructs and harvested in NP-40 lysis buffer (10 mM Tris-HCl pH 7.5, 137 mM NaCl, 1 mM EDTA, 10 % glycerol, 0.5 % NP-40, 80 mM β-glycerophosphate, 1 mM Na_3_VO_4_, 10 mM NaF, and protease inhibitor cocktail). Protein extracts (0.1–1 mg) were incubated with either anti-FLAG M2 agarose beads (Sigma-Aldrich) for 2 h or anti-myc (9E10) agarose beads (Santa Cruz Biotechnology) overnight at 4 °C with rotation. Immunoprecipitates were washed four times with lysis buffer before elution by boiling in Laemmli sample buffer.

Protein complexes were resolved by SDS-PAGE and transferred to PVDF membranes for immunoblotting. Membranes were probed with primary antibodies against FLAG (M2, Sigma-Aldrich), myc (9E10, Santa Cruz Biotechnology), or PER2 (Sigma-Aldrich), followed by horseradish peroxidase-conjugated anti-rabbit or anti-mouse IgG secondary antibodies (Cell Signaling Technology). Immunoreactive bands were visualized using SuperSignal West Pico chemiluminescent substrate (Pierce).

### 2.4 *In vitro* binding assays

*In vitro* transcription and translation of pCS2+(myc)_6_-PER2, (myc)_6_-BRCA1 (residues 1–400), (myc)_6_-BRCA1 (residues 1670–1863), and FLAG-POU domain of POU2F1(OCT-1) were performed using the SP6 high-yield TNT coupled reticulocyte lysate system (Promega) according to the manufacturer's instructions. Recombinant (myc)_6_-PER2, (myc)_6_-BRCA1 (1–400), and (myc)_6_-BRCA1 (1670–1863) proteins were radiolabeled with [^35^S]-methionine during translation.

For binding assays, recombinant FLAG-POU domain alone or in combination with (myc)_6_-BRCA1 (1–400) or (myc)_6_-BRCA1 (1670–1863) was first incubated with anti-FLAG M2 agarose beads for 1 h at 4 °C, followed by addition of [^35^S]-labeled (myc)_6_-PER2 and incubation for an additional 2 h. Beads were washed extensively with binding buffer, and bound proteins were eluted by boiling in Laemmli buffer, resolved by SDS-PAGE, and visualized by autoradiography.

### 2.5 GST-fused protein design and purification

For PER2 and BRCA1, fragment boundaries were positioned to avoid truncating predicted α-helical regions while capturing functionally annotated domains (in each case, residues are indicated within parenthesis). Specifically: (i) PER2(1–172), N-terminal region preceding the PAS-A domain, predicted to be largely disordered and containing regulatory phosphorylation sites, (ii) PER2(173–355), PAS-A domain (residues ∼200–270) and flanking sequences, representing a structured domain with predicted α-helical content involved in protein-protein interactions, (iii) PER2(356–574), region immediately C-terminal to PAS-A, encompassing the PAS-B domain (residues ∼290–390) and adjacent sequences, (iv) PER2(575–682), linker region between PAS-B and the CK1δ/*ε* regulatory domain, predicted to be intrinsically disordered and containing multiple phosphorylation sites and nuclear export signals, (v) PER2(683–872), CK1δ/*ε* regulatory domain containing the FASP motif (residues ∼755–795) and adjacent sequences (fragment boundaries were positioned to avoid truncating predicted α-helical regions flanking the regulatory domain), (vi) PER2(873–1120), central region predicted to contain mixed structured and disordered segments, including nuclear localization signals and protein interaction motifs and, (vii) PER2(1121–1255), C-terminal region encompassing the CRY-binding domain (residues ∼1150–1240), which mediates critical circadian clock feedback regulation. For BRCA1 domain mapping, boundaries were as follows: (i) BRCA1 (1–178), minimal N-terminal fragment containing the RING domain (residues ∼1–103), which forms a structured zinc-binding module essential for E3 ubiquitin ligase activity, (ii) BRCA1 (1–333) and (iii) BRCA1 (1–400), extended N-terminal constructs capturing the RING domain and progressively more of the adjacent disordered region, allowing assessment of whether extended sequences enhance binding affinity or specificity, (iv) BRCA1 (1–500), includes RING domain and the complete serine-rich region, (v) BRCA1 (852–1379), central region encompassing the coiled-coil domain (residues ∼1390–1424 are just outside this fragment), predicted to adopt α-helical secondary structure, (vi) BRCA1 (1670–1770) and (vii) BRCA1 (1670–1863), C-terminal constructs containing the tandem BRCT domains (BRCT1: residues ∼1650–1736; BRCT2: residues ∼1760–1855). The BRCT domains form a structured tandem repeat with well-defined α/β topology. The fragment boundaries were positioned to preserve the complete BRCT fold: residue 1670 begins upstream of BRCT1 to include the inter-domain linker, while residue 1863 extends beyond BRCT2 to ensure the complete structured domain is captured.

GST fusion proteins were expressed in *Escherichia coli* BL21 (DE3) cells induced with 0.1 mM isopropyl β-D-1-thiogalactopyranoside (IPTG) for 4 h at 30 °C. Bacterial pellets were lysed by sonication, and GST-tagged proteins were purified using glutathione-Sepharose 4B chromatography (Cytiva) according to the manufacturer's instructions. When indicated, proteins were cleaved from the GST tag using thrombin (10 U/μL, Sigma-Aldrich) at 4 °C overnight. Cleaved proteins were further purified by size exclusion chromatography using a HiLoad 16/60 Superdex 75 prep-grade column (Cytiva) equilibrated in 20 mM Tris-HCl pH 7.4, 100 mM NaCl, 5 mM DTT. Protein concentration was determined using the Bradford protein assay (Bio-Rad).

### 2.6 Protein pull-down assays

For pull-down assays, 20 μg of GST fusion proteins immobilized on glutathione-Sepharose beads, or an equivalent volume of glutathione beads alone (GST control), were incubated with 3 μL of *in vitro* translated, [^35^S]-methionine-labeled (myc)_6_-POU domain, (myc)_6_-PER2, or FLAG-BRCA1 in binding buffer (20 mM Tris-HCl pH 7.4, 100 mM NaCl, 5 mM EDTA, 0.1 % Triton X-100) for 1 h at 4 °C with rotation. Beads were washed sequentially with low-salt (100 mM NaCl) and high-salt (1 M NaCl) binding buffers. Bound proteins were eluted by boiling in Laemmli buffer, resolved by SDS-PAGE, and visualized by autoradiography.

### 2.7 Luciferase reporter assays

A fragment of the human *ESR1* promoter spanning nucleotides −255 to +144 relative to the transcription start site was amplified by PCR and cloned into the KpnI and NheI restriction sites of the pGL2-Basic luciferase reporter vector (Promega). For reporter assays, CHO and MCF-7 cells were seeded in 12-well plates at 5 × 10^4^ cells per well and co-transfected with 200 ng pGL2-*ESR1* reporter plasmid and increasing amounts (50–200 ng) of pCS2+(myc)_6_-POU, pCS2+(myc)_6_-BRCA1, and pCS2+(myc)_6_-PER2 expression vectors. The pCMV-β-galactosidase plasmid (100 ng) was co-transfected to normalize for transfection efficiency. Total DNA (1 μg per well) was kept constant by adding empty pCS2+(myc)_6_ vector as needed.

Cells were harvested 24 h (CHO) or 48 h (MCF-7) post-transfection and lysed in 1X cell lysis buffer (Promega). Luciferase activity was measured using the Bright-Glo Luciferase Assay System (Promega) on a GloMax luminometer (Promega) according to the manufacturer's protocol. Relative luciferase activity was normalized to β-galactosidase activity, which was determined using the o-nitrophenyl-β-D-galactopyranoside (ONPG) colorimetric assay. All experiments were performed in triplicate and repeated at least three times.

### 2.8 Electrophoretic mobility shift assays (EMSA)

A double-stranded DNA probe containing the POU2F1(OCT-1)-binding element located at positions −56 to −42 relative to the human *ESR1* transcription start site was generated by annealing complementary oligonucleotides: 5′-GCTATGGCCTATGCATATGAAGCCTTTATT-3’ (forward) and 5′-AATAAAGGCTTCATATGCATAGGCCATAGC-3’ (reverse). Oligonucleotides were annealed by heating to 95 °C for 5 min followed by slow cooling to room temperature overnight. The annealed probe was 5′-end-labeled with [γ-^32^P]-ATP using T4 polynucleotide kinase (New England Biolabs) and purified using NucAway spin columns (Ambion).

Binding reactions were performed by incubating recombinant proteins with radiolabeled probe (∼10,000 cpm) for 20 min at room temperature in binding buffer (20 mM HEPES pH 7.9, 2 mM MgCl_2_, 10 % glycerol, 2 mM DTT, 36 ng poly (dI·dC)). Protein-DNA complexes were resolved by electrophoresis on 5 % non-denaturing polyacrylamide gels in 0.5 × Tris-borate-EDTA (TBE) buffer at 100 V for 2 h at 4 °C. Gels were dried and visualized by autoradiography.

### 2.9 Chromatin immunoprecipitation (ChIP) assays

Chromatin immunoprecipitation assays were performed as previously described with modifications (de Jonge et al., 2020). Briefly, cells (8 × 10^6^) were transfected with pCS2+FLAG-POU, pCS2+(myc)_6_-PER2, pCS2+(myc)_6_-BRCA1, or pCS2+(myc)_6_-POU expression vectors as indicated in the figure legends and cultured for 24 h prior to cross-linking. Cross-linking was performed with 1 % formaldehyde for 15 min at room temperature and quenched by addition of glycine to a final concentration of 125 mM for 5 min.

Cross-linked cells were harvested by scraping in ice-cold phosphate-buffered saline (1xPBS) containing protease inhibitors (1 μM phenylmethylsulfonyl fluoride, 0.3 μM aprotinin, 10 μM leupeptin, and 1 μM pepstatin A). Cell pellets were lysed in SDS lysis buffer (1 % SDS, 10 mM EDTA, 50 mM Tris-HCl pH 8.0) on ice for 10 min. Chromatin was sheared by sonication using a probe-tipped sonicator (Branson Sonifier 450) with 20 cycles of 30 s pulses at 95 % amplitude, separated by 30 s cooling intervals on ice, to generate DNA fragments of 200–500 bp. Sonicated lysates were cleared by centrifugation and supernatants were transferred to clean tubes.

For immunoprecipitation, sonicated lysates were diluted 10-fold in ChIP dilution buffer (0.01 % SDS, 1.1 % Triton X-100, 1.2 mM EDTA, 16.7 mM Tris-HCl pH 8.0, 167 mM NaCl) supplemented with protease inhibitors as described above, yielding a final volume of 2.5 ml per sample. A portion of each lysate (1 % of total volume) was reserved as input DNA control. The remaining lysate was subjected to immunoprecipitation with the appropriate antibody/bead conjugate. Anti-FLAG M2 agarose beads (10 μl of 1:1 slurry, Sigma-Aldrich) were used for FLAG-tagged proteins. For negative controls, 1 μg of normal mouse IgG antibody (Biocare Medical) was pre-bound to 20 μl protein A agarose beads (Abcam). For positive controls, 1 μg of anti-RNA polymerase II antibody (Sigma-Aldrich) was pre-bound to 20 μl protein A agarose beads. All immunoprecipitations were performed for 2 h at 4 °C with rotation. Following immunoprecipitation, beads were pelleted by brief centrifugation and washed sequentially with the following buffers (5 min per wash at 4 °C with rotation): low-salt wash buffer (0.1 % SDS, 1 % Triton X-100, 2 mM EDTA, 20 mM Tris-HCl pH 8.0, 150 mM NaCl), high-salt wash buffer (0.1 % SDS, 1 % Triton X-100, 2 mM EDTA, 20 mM Tris-HCl pH 8.0, 500 mM NaCl), LiCl wash buffer (0.25 M LiCl, 1 % NP-40, 1 % sodium deoxycholate, 1 mM EDTA, 10 mM Tris-HCl pH 8.0), and TE buffer (10 mM Tris-HCl pH 8.0, 1 mM EDTA, two washes).

Protein-DNA complexes were eluted from beads by adding 200 μl of elution buffer (1 % SDS, 0.1 M NaHCO_3_) and incubating at room temperature for 15 min with vortexing. Both immunoprecipitated samples and input DNA were adjusted to 200 mM NaCl and incubated at 65 °C for 4 h (or overnight) to reverse cross-links. Samples were then treated with RNase A (0.2 mg/ml) for 30 min at 37 °C, followed by proteinase K (0.2 mg/ml) digestion for 2 h at 55 °C. DNA was extracted using phenol:chloroform:isoamyl alcohol (25:24:1, v/v/v) followed by ethanol precipitation as previously described (Butler, 2012). Purified DNA was resuspended in 50 μL TE buffer and analyzed by PCR or quantitative PCR (qPCR) using primers flanking the POU2F1(OCT-1)-binding site in the *ESR1* promoter region.

### 2.10 Far-UV circular dichroism

Far-UV circular dichroism (CD) spectra were acquired at 23 °C using a Jasco J-815 spectropolarimeter equipped with a Peltier temperature controller. Protein samples (10 μM) were prepared in CD buffer (5 mM Tris-HCl pH 8.0, 100 mM kF, 0.1 mM DTT). Spectra were collected in a 1-mm path-length quartz cuvette from 260 to 195 nm with a bandwidth of 1 nm, response time of 1 s, and scan speed of 20 nm/min. Each spectrum represents the average of five accumulated scans. Buffer baseline spectra were subtracted from protein spectra to correct for background signal. Secondary structure content was estimated by spectral deconvolution using the DichroWeb online server with the CONTIN algorithm. The normalized root mean square deviation (NRMSD) between experimental and calculated spectra was used to assess fit quality.

### 2.11 Multimeric complex predictions

Multimeric protein complex predictions were performed using the AlphaPulldown 2.0 pipeline (Molodenskiy et al., 2025). Precomputed protein features were obtained from AlphaPulldown's database for *Homo sapiens* proteins: PER2 (UniProt ID: O15055), BRCA1 (UniProt ID: P38398), and POU2F1 (OCT-1, UniProt ID: P14859). Protein sequences were retrieved in their native format and used without additional preprocessing. Complex modeling was performed using AlphaPulldown with the multimer function and default parameters (5 models per complex, 3 recycling iterations). Models were ranked according to AlphaPulldown's internal ranking system based on predicted local distance difference test (pLDDT) and predicted aligned error (PAE) scores. The highest-ranked model (rank 0) was selected for each complex for subsequent structural analysis.

### 2.12 Structural modeling and protein-protein interface analysis

Predicted structures of full-length PER2 in complex with BRCA1, a PER2 fragment (residues 356–574) in complex with a BRCA1 fragment (residues 1670–1863), and full-length PER2 in complex with POU2F1(OCT-1) were analyzed using models generated by AlphaPulldown as described above.

Model confidence was validated by confirming that per-residue predicted local distance difference test (pLDDT) scores were correctly encoded in the B-factor field of the output PDB files. This was accomplished by correlating pLDDT values from the confidence model multimer JSON file, also generated by AlphaPullDown, with B-factor values in the corresponding PDB file. The resulting correlation was highly positive (R = 0.99), validating the use of B-factor values as a proxy for pLDDT scores. Regions with pLDDT scores below 60 were classified as low confidence and were either hidden or rendered with transparency, while high-confidence regions (pLDDT ≥60) were retained for analysis and visualization using PyMOL v. 3.1.6.1 (Schrödinger, LLC). We additionally evaluated predicted alignment error (PAE) scores to assess confidence in the relative positioning of protein domains and interaction interfaces. Only regions meeting both high pLDDT (≥60) and low PAE criteria were interpreted as structurally meaningful interfaces and used for contact analysis.

To define potential binding interfaces, we developed a custom Python script that identifies and records one representative atomic contact per interacting residue pair across four interaction classes: hydrogen bonds, salt bridges, hydrophobic contacts, and steric clashes. The script identifies heavy-atom pairs within user-specified distance cutoffs: 3.6 Å for hydrogen bonds (with a maximum angle deviation of 55° from linearity), 4.0 Å for salt bridges and hydrophobic contacts, and 2.2 Å for steric clashes. For each residue pair meeting these criteria, the script calculates the shortest inter-atomic distance and generates both a CSV-formatted summary table and labeled distance objects in PyMOL tagged by interaction type. This analysis was applied to the PER2 fragment:BRCA1 fragment complex, the full-length PER2:BRCA1 complex, and the full-length PER2:POU2F1(OCT-1) complex, enabling comprehensive characterization of residue-level contacts and visualization of high-confidence protein-protein interactions.

### 2.13 Circadian gene expression analysis

Circadian gene expression datasets were obtained from the National Center for Biotechnology Information (NCBI) Gene Expression Omnibus (GEO) database. Three independent datasets were selected to assess circadian gene expression patterns in wild-type and circadian clock-deficient mouse models. For wild-type circadian profiling across multiple tissues, dataset GSE54652 was used, which contains transcriptome data from 12 mouse tissues including white adipose, liver, and kidney tissues (Zhang et al., 2014a). Dataset GSE11923 was additionally used to validate circadian expression patterns in liver tissue from C57BL/6J mice (Hughes et al., 2009). Expression data for GSE54652 and GSE11923 were retrieved using the GEO2R integrated analysis tool.

To investigate the functional role of core circadian clock components, dataset GSE171975 was utilized for comparative analysis between wild-type and circadian clock-deficient mice (Aviram et al., 2021). This dataset contains liver expression profiles of samples collected from wild-type (WT, C57BL/6) and *Per1* and *Per2* double knockout (*Per1*^−/−^/*Per2*^−/−^, Per1/2 dKO) mice on the C57BL/6 background (Zheng et al., 2001). Processed expression data for GSE171975 were downloaded directly from the GEO supplementary files. All circadian rhythm analyses were performed using meta2d function from MetaCycle R package, which integrates three established algorithms: ARSER (ARS), JTK_CYCLE (JTK), and Lomb-Scargle (LS) for rhythm detection (Wu et al., 2016). All datasets were analyzed using pre-processed expression values as provided in GEO or extracted via GEO2R, without additional normalization. For each gene, key circadian parameters were computed: period, phase, amplitude and baseline expression level. Genes with integrated P < 0.01 were classified as significantly rhythmic.

## 3 Results

### 3.1 The core clock component PER2 directly interacts with the breast cancer type 1 susceptibility protein (BRCA1)

To identify previously uncharacterized PER2-interacting proteins, we performed a bacterial two-hybrid screen using a human liver cDNA library. We chose to use a liver cDNA library because the liver is one of the most transcriptionally active organs, displaying extensive circadian regulation of metabolic, detoxification, and biosynthetic pathways (Zhang et al., 2014b; Panda, 2016). Moreover, clock-controlled transcriptional programs in the liver drive rhythmic expression of thousands of genes, underscoring the tissue's utility for identifying clock-regulated molecular interactions thus, increasing the likelihood of capturing biologically meaningful PER2 partners (Koike et al., 2012; Atger et al., 2015).

Four PER2 constructs were used as baits: full-length PER2 (comprising residues 1 to 1255) and three fragments spanning residues 356–574, 683–872, and 822–1255, designated PER2(356–574), PER2(683–872), and PER2(822–1255), respectively. These fragments were selected to represent distinct functional domains of PER2, including the PAS and casein kinase 1 binding domains, and C-terminal regulatory regions.

The initial screen yielded 120 candidate interactors (67 strong, 53 weak) based on growth on non-selective medium containing tetracycline and chloramphenicol. To eliminate false positives, candidates were subjected to stringent secondary selection on medium supplemented with 5 mM 3-amino-1,2,4-triazole (3-AT), which inhibits the *HIS3* gene product and requires stronger transcriptional activation for bacterial survival. In the BacterioMatch II system, authentic protein-protein interactions between bait and prey activate transcription of two reporter genes: *HIS3*, conferring 3-AT resistance, and *aadA*, conferring streptomycin resistance. Only clones forming stable, functional protein complexes survive dual selection. Following this validation, 90 positive clones were confirmed and sequenced.

**Fig. 1:**
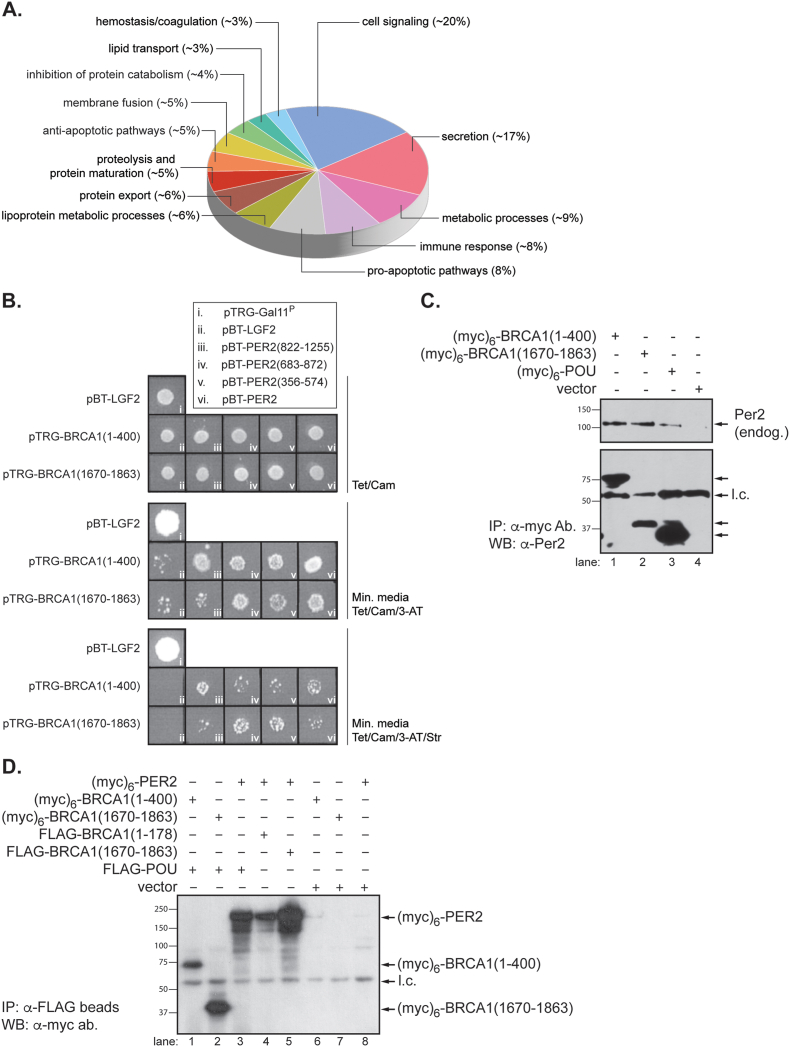
**PER2 physically interacts with the N- and C-terminal domains of BRCA1.** (**A**) Gene Ontology classification of PER2-interacting proteins identified by bacterial two-hybrid screening. Ninety validated positive clones were functionally classified, revealing PER2 interactions across diverse biological processes. T (**B**) Bacterial two-hybrid interaction assay confirming direct interaction between PER2 and BRCA1 fragments. Bacteria co-transformed with bait plasmids encoding full-length human PER2 or PER2 fragments (residues 822–1255, 683–872, or 356–574) and prey plasmids encoding BRCA1 fragments (residues 1–400 or 1670–1863). Following initial selection on non-selective medium (tetracycline/chloramphenicol, Tet/Cam), candidate interactors were subjected to stringent validation on medium containing 3-amino-1,2,4-triazole (Tet/Cam/3-AT) or 3-AT plus streptomycin (Tet/Cam/3-AT/Str) to confirm the strongest protein-protein interactions. The PER2(356–574) fragment shows particularly strong interactions with both BRCA1 domains. Positive control (pBT-LGF2/pTRG-Gal11P) and negative controls (empty pTRG vector) validate assay specificity. (**C**) Co-immunoprecipitation of endogenous PER2 with myc-tagged BRCA1 fragments in CHO cells. Immunoprecipitation with anti-myc beads followed by immunoblotting with anti-Per2 antibody demonstrates that endogenous mouse Per2 (Per2) associates with (myc)_6_-BRCA1 (1–400) and (myc)_6_-BRCA1 (1670–1863), but not with empty vector control. Co-precipitation with (myc)_6_-POU DNA-binding domain was included and serves as a positive control. The heavy IgG chain band serves as loading control (l.c.). These results validate mammalian cell-based interactions at endogenous protein expression levels. (**D**) Co-immunoprecipitation in human MCF-7 breast cancer cells demonstrating PER2, BRCA1, and POU DNA-binding domain interactions. MCF-7 cells were co-transfected with myc-tagged PER2 and FLAG-tagged BRCA1 fragments (residues 1–178, 1–400, or 1670–1863) or FLAG-tagged POU DNA-binding domain. Immunoprecipitation with anti-FLAG beads followed by immunoblotting with anti-myc antibody shows that (myc)_6_-PER2 co-immunoprecipitates with FLAG-BRCA1 fragments and FLAG-POU. Notably, the minimal BRCA1 fragment (1–178) is sufficient for PER2 interaction, establishing a discrete N-terminal binding interface. No co-immunoprecipitation occurs with empty vector, confirming interaction specificity.

Functional classification of the 90 validated interactors using Gene Ontology annotations revealed representation across diverse biological processes (Fig. 1A). The most abundant categories included cell signaling (∼20 %), secretion (∼17 %), metabolic processes (∼9 %), pro-apoptotic pathways (∼8 %), immune response (∼8 %), protein export (∼6 %), lipoprotein metabolism (∼6 %), along with smaller representations of proteolysis and protein maturation (∼5 %), membrane fusion (∼5 %), anti-apoptotic pathways (∼5 %), lipid transport (∼3 %), and hemostasis/coagulation (∼3 %) (Fig. 1A). This diversity reflects the multifunctional nature of PER2 beyond its canonical role in circadian timekeeping.

Among the validated candidates, we identified multiple independent clones encoding fragments of BRCA1, a well-established tumor suppressor with critical roles in DNA damage repair, transcriptional regulation, and genome stability. Importantly, both N-terminal (residues 1–400) and C-terminal (residues 1670–1863, comprising the tandem BRCT domains) fragments of BRCA1 were recovered as PER2 interactors, suggesting the presence of multiple independent binding interfaces between these proteins.

To confirm the specificity of PER2:BRCA1 interactions, we performed direct co-transformation experiments using defined bait and prey constructs. The four PER2 constructs described above were cloned into the pBT bait vector, while BRCA1 fragments encoding residues 1–400 (N-terminal domain) and 1670–1863 (BRCT domain) were cloned into the pTRG prey vector. Co-transformants were plated on media of increasing selective stringency: dual selection (tetracycline/chloramphenicol), triple selection (tetracycline/chloramphenicol/3-AT), and quadruple selection (tetracycline/chloramphenicol/3-AT/streptomycin) (Fig. 1B). Robust colony growth was observed for all PER2 constructs co-transformed with BRCA1 fragment under triple selection conditions, confirming HIS3 reporter activation. Furthermore, growth on quadruple selection medium confirmed activation of the *aadA* reporter, providing independent verification of genuine protein-protein interactions. Notably, the PER2(356–574) fragment, which encompasses the region immediately C-terminal to the PAS A and B domains, exhibited particularly strong interactions with both BRCA1 regions, as evidenced by enhanced colony growth under the most stringent selection conditions. These bacterial two-hybrid results identify BRCA1 as a novel PER2-interacting protein and suggest that PER2 engages BRCA1 through at least two distinct binding interfaces involving both the N-terminal and C-terminal regions of BRCA1.

To validate these interactions under physiologically relevant conditions, we performed co-immunoprecipitation experiments using different mammalian cell lines. CHO cells were transiently transfected with (myc)_6_-BRCA1 (1–400) representing the N-terminal domain, or (myc)_6_-BRCA1 (1670–1863) containing the C-terminal BRCT domains of BRCA1. Immunoprecipitation with anti-myc antibody followed by immunoblotting with anti-Per2 revealed that endogenous Per2 associates with both BRCA1 fragments (Fig. 1C). This indicates that the PER2:BRCA1 interaction is specific and occurs at endogenous protein levels.

To extend these findings to a human cell system, we performed reciprocal co-immunoprecipitation experiments in MCF-7 cells, an ER-positive human breast cancer cell line. Cells were co-transfected with myc-tagged full-length PER2 and FLAG-BRCA1 constructs, including FLAG-BRCA1 (1–178) and FLAG-BRCA1 (1670–1863). (myc)_6_-PER2 co-immunoprecipitated with both BRCA1 fragments (Fig. 1D). Of note, the minimal N-terminal BRCA1 fragment spanning amino acids 1–178 was sufficient to interact with full-length PER2, suggesting that a discrete binding interface exists within the N-terminal 178 amino acids of BRCA1. Collectively, the bacterial complementation and co-immunoprecipitation assay results identify BRCA1 as a previously uncharacterized binding partner of PER2 and suggest that they interact through at least two distinct binding interfaces involving both the N-terminal and C-terminal regions of BRCA1.

### 3.2 PER2 and BRCA1 interact through multiple discrete binding interfaces

**Fig. 2:**
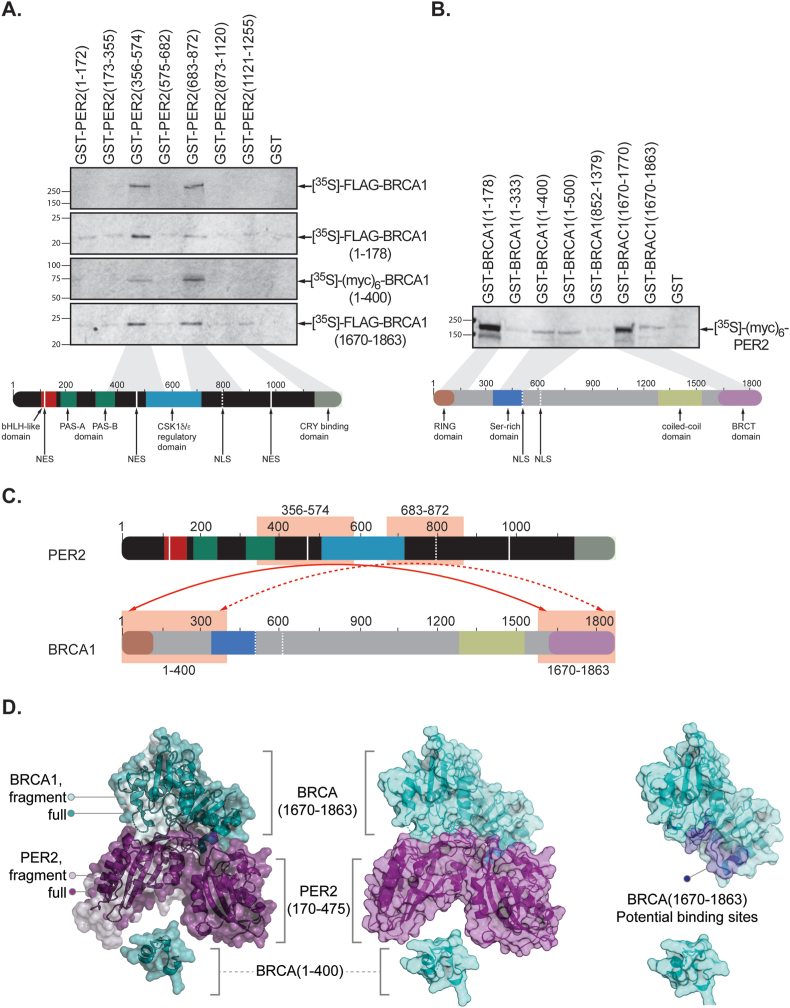
**Domain mapping and structural modeling reveal multiple interaction regions between PER2 and BRCA1.** (**A**) GST pull-down assay systematically mapping PER2:BRCA1 interaction domains. Upper panel: Seven GST-tagged PER2 fragments spanning the entire protein (residues 1–172, 173–355, 356–574, 575–682, 683–872, 873–1120, and 1121–1255) were incubated with [^35^S]-radiolabeled BRCA1 constructs [full-length FLAG-BRCA1, FLAG-BRCA1 (1–178), (myc)_6_-BRCA1 (1–400), and FLAG-BRCA1 (1670–1863)]. Following pull-down on glutathione beads, bound complexes were resolved by SDS-PAGE and visualized by autoradiography. Full-length BRCA1 and all truncated BRCA1 fragments bind specifically to PER2 fragments 356–574 and 683–872, with no detectable binding to other PER2 regions. Lower panel: Schematic representation of PER2 domain organization with mapped BRCA1-binding regions highlighted, showing positions relative to PAS domains, CK1δ/*ε* regulatory domain, nuclear localization/export signals (NLS/NES), and CRY-binding domain. (**B**) Reciprocal GST pull-down assay mapping BRCA1 domains that interact with PER2. Upper panel: Seven GST-tagged BRCA1 fragments (residues 1–178, 1–333, 1–400, 1–500, 852–1379, 1670–1770, and 1670–1863) were incubated with [^35^S]-radiolabeled (myc)_6_-PER2. Following pull-down on glutathione beads, bound complexes were resolved and visualized. Full-length (myc)_6_-PER2 interacts with N-terminal constructs (residues 1–400, 1–500) and C-terminal BRCT domain constructs (residues 1670–1770, 1670–1863), including the minimal fragment (1–178). The middle region (residues 852–1379) shows weak or no binding. Lower panel: Schematic showing BRCA1 domain organization (RING domain, Ser-rich region, coiled-coil domain, and BRCT domains) with mapped PER2-binding regions, establishing that both BRCA1 termini interact with at least two discrete PER2 regions. (**C**) Representative model summarizing key findings: PER2 binds BRCA1 through multiple discrete interfaces, PER2 regions 356–574 and 683–872 interact with both the N-terminal (1–400) region and the C-terminal (BRCT domains (1670–1863) of BRCA1, as validated by bacterial two-hybrid assays, GST pull-down, co-immunoprecipitation, and Alphapulldown modeling. (**D**) AlphaPulldown structural comparison of PER2:BRCA1 complexes. Only these high-confidence regions (pLDDT ≥60; low PAE), labeled as BRCA1 (1–400) and (1670–1863) as well as PER2 (170–475), were used to interpret interaction interfaces. Low-confidence regions (pLDDT <60) are shown with transparency or hidden in structural representations and were not used to infer specific protein-protein contacts. Left: Overlay of high-confidence regions from full-length BRCA1:PER2 complex (teal and purple surfaces, respectively) and fragment-based models (BRCA1 residues 1670–1863 in grey-teal; PER2 residues 170–475 in grey-purple), showing consistent positioning and interaction geometry. Middle: Interaction interface regions of the full-length, high-confidence PER2:BRCA1 complex. Right: Detailed view of the binding contact region between BRCA1 BRCT domain (residues 1655–1839) and PER2. Structural modeling predicted 361 residue contacts, defined as residues within 4 Å, between PER2 and BRCA1.

Having validated PER2:BRCA1 interactions, we next sought to map the precise interacting domains between these proteins using GST pull-down assays with purified recombinant proteins (Fig. 2A). Seven GST-tagged PER2 fragments were generated with boundaries designed based on predicted secondary structure and domain annotations to preserve structural integrity: GST-PER2(1–172) (N-terminal disordered region), GST-PER2(173–355) (PAS-A domain and flanking sequences), GST-PER2(356–574) (PAS-B domain region), GST-PER2(575–682) (disordered linker with nuclear export signal), GST-PER2(683–872) (Casein Kinase 1δ/*ε* regulatory domain), GST-PER2(873–1120) (central region with nuclear localization signal), and GST-PER2(1121–1255) (CRY-binding domain) (Fig. 2A, lower panel). Fragment boundaries were positioned to avoid truncating predicted α-helical regions while capturing functionally annotated domains.

Each GST-PER2 fragment was incubated with [^35^S]-methionine-labeled BRCA1 constructs: full-length FLAG-BRCA1 (residues 1–1863, named FLAG-BRCA1), a minimal N-terminal fragment FLAG-BRCA1 (1–178), an extended N-terminal construct (myc)_6_-BRCA1 (1–400), and a C-terminal fragment containing the tandem BRCT domains FLAG-BRCA1 (1670–1863) (Fig. 2A). Full-length FLAG-BRCA1 and (myc)_6_-BRCA1 (1–400) exhibited similar binding to GST-PER2(356–574) and GST-PER2(683–872), with no detectable binding to other PER2 fragments. Remarkably, the minimal N-terminal fragment FLAG-BRCA1 (1–178) also bound to these same PER2 regions, indicating that amino acids 1–178 contain a functionally competent PER2-binding interface. Similarly, the C-terminal BRCT domain construct FLAG-BRCA1 (1670–1863) interacted with PER2(356–574) and PER2(683–872), establishing that both termini of BRCA1 independently recognize at least two discrete regions within PER2.

To map PER2-binding domains within BRCA1, we performed reciprocal pull-down experiments using GST-tagged BRCA1 fragments and [^35^S]-labeled (myc)_6_-tagged full-length PER2 (Fig. 2B). Seven GST-tagged fragments were generated: GST-BRCA1 (1–178) (RING domain), GST-BRCA1 (1–333), GST-BRCA1 (1–400), GST-BRCA1 (1–500) (N-terminal extensions), GST-BRCA1 (852–1379) (central coiled-coil region), and GST-BRCA1 (1670–1770), GST-BRCA1 (1670–1863) (tandem BRCT domains) (Fig. 2B). BRCT domain boundaries (1670–1863) were selected based on known BRCT structures to preserve the complete tandem α/β fold and inter-domain linker, ensuring proper domain folding. Full-length (myc)_6_-PER2 bound to multiple BRCA1 fragments, including the N-terminal constructs GST-BRCA1 (1–400) and GST-BRCA1 (1–500), as well as the C-terminal BRCT domain constructs GST-BRCA1 (1670–1770) and GST-BRCA1 (1670–1863). Importantly, even the minimal GST-BRCA1 (1–178) fragment was sufficient to pull down full-length PER2, confirming that a discrete PER2-binding interface resides within the first 178 amino acids of BRCA1. The middle region, GST-BRCA1 (852–1379), showed weak or no binding, suggesting that the primary PER2-binding sites are localized to the N- and C-terminal regions of BRCA1.

**Fig. 3:**
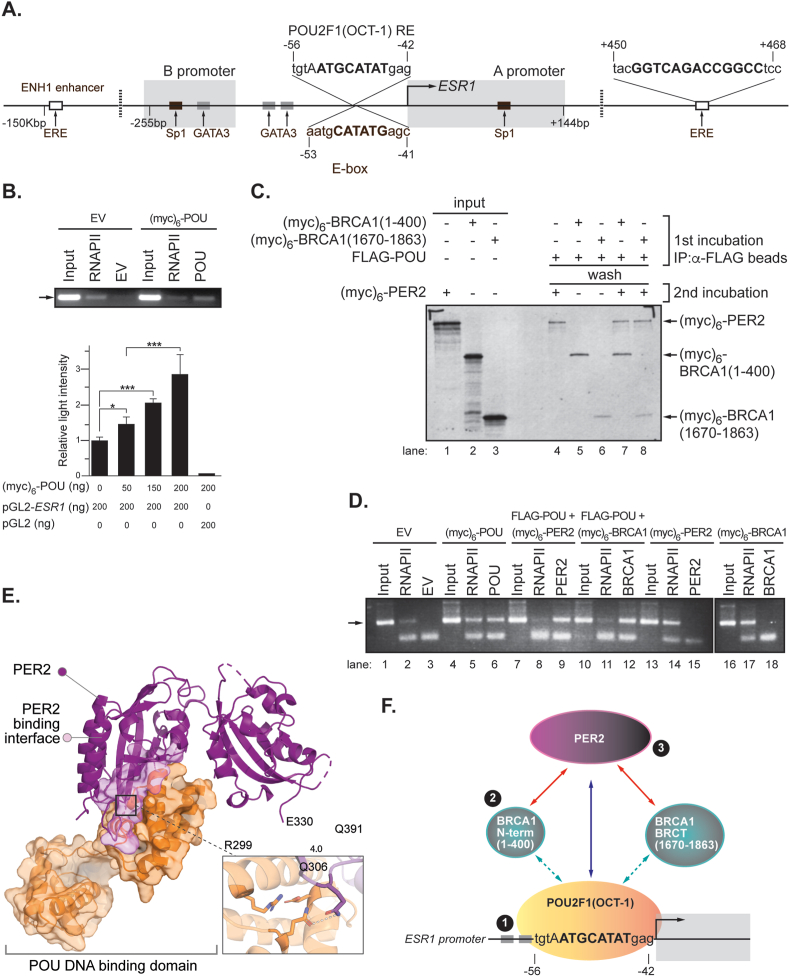
**PER2 interacts with the DNA-binding domain of POU2F1(OCT-1) and form a ternary complex that converges at the *ESR1* promoter.** (**A**) Schematic of the human *ESR1* promoter showing the regulatory region utilized in ChIP and reporter assays (−255 to +144 bp relative to transcription start site). This region contains multiple regulatory elements including GATA3 and Sp1 binding sites, and critically, a near-canonical POU2F1(OCT-1) response element (5′-ATGCAAAT-3′) located at positions −56 to −42 bp, which overlaps with a non-canonical E-box element (−53 to −41 bp). The promoter contains two tissue-specific promoters (A and B) that drive ERα expression in breast epithelial cells. (**B**) Upper panel: Chromatin immunoprecipitation assay demonstrating POU DNA-binding domain interaction with the *ESR1* promoter. MCF-7 cells were transfected with myc-tagged POU DNA-binding domain or empty vector. Cross-linked chromatin was immunoprecipitated with anti-myc antibody, and the *ESR1* promoter region containing the POU response element was amplified by PCR. The POU domain shows robust enrichment compared to empty vector. Input chromatin (10 %) and RNA polymerase II ChIP serve as loading and positive controls, respectively. Lower panel: Luciferase reporter assay measuring *ESR1* promoter transcriptional activity. Cells were co-transfected with pGL2-*ESR1* promoter reporter (200 ng), β-galactosidase control (100 ng), and increasing amounts of (myc)_6_-POU DNA-binding domain expression vector (0, 50, 150, or 200 ng). POU domain expression results in dose-dependent activation of the *ESR1* promoter, with maximal activation (∼3-fold) at 200 ng ∗∗∗p < 0.001 by Student's t-test. (**C**) *In vitro* binding assay demonstrating simultaneous recruitment of PER2, BRCA1, and POU DNA binding domain into a ternary complex. BRCA1 fragments (residues 1–400 or 1670–1863) and full-length PER2 were *in vitro* transcribed, translated, and radiolabeled with [^35^S]-methionine. Radiolabeled proteins were incubated sequentially with FLAG-POU immobilized on anti-FLAG beads: first with BRCA1 fragments to form a binary complex, followed by extensive washing and incubation with PER2. Bound complexes were eluted and resolved by SDS-PAGE and autoradiography. Both BRCA1 fragments remain associated with POU DNA binding-domain after addition of PER2, demonstrating stable ternary complex formation. (**D**) Chromatin immunoprecipitation assay showing POU-dependent recruitment of PER2 and BRCA1 to the *ESR1* promoter. HEK293T cells were transfected with (myc)_6_-POU DNA binding-domain alone, FLAG-POU with (myc)_6_-PER2, FLAG-POU with (myc)_6_-BRCA1, or (myc)_6_-PER2 or (myc)_6_-BRCA1 alone. Cross-linked chromatin was immunoprecipitated with anti-myc or anti-FLAG antibodies, and the *ESR1* promoter region was amplified by PCR. Neither PER2 nor BRCA1 shows binding to the promoter when expressed alone; however, both are robustly recruited when co-expressed with POU, demonstrating that POU DNA binding is required for their chromatin recruitment. (**E**) Structural features of the PER2:POU DNA-binding domain complex predicted by AlphaPulldown are based exclusively on high-confidence regions (pLDDT ≥60 and low PAE) that are suitable for structural interpretation. Left: High-confidence region of the PER2:POU complex with PER2 shown in purple (cartoon representation) and the POU DNA-binding domain shown in orange (surface and cartoon representations). Right (inset): Predicted binding interface residues on POU include R299, E330, and Q306, which cluster near the DNA recognition helix, along with additional contacts involving PER2 residues. These residues are conserved across vertebrates and positioned at the protein-DNA interface, highlighting their functional importance. (**F**) Cartoon representation of the PER2:BRCA1:POU2F1(OCT-1) ternary complex interactions at the *ESR1* promoter. Solid lines indicate PER2 interactions with BRCA1 (red) and POU2F1(OCT-1) (blue). Dashed teal lines indicate BRCA1:POU2F1(OCT-1) interactions. (1) POU2F1 binds DNA, (2) BRCA1 serves as a molecular scaffold, and (3) PER2 recruitment provides circadian gating.

These bidirectional pull-down experiments establish that PER2 and BRCA1 interact through at least two independent binding interfaces: (i) the N-terminal region of BRCA1 (residues 1–400) interacts with PER2 regions 356–574 and 683–872, and (ii) the C-terminal BRCT domains of BRCA1 similarly interact with these same PER2 regions (Fig. 2C). While these fragment-based studies provide essential mechanistic insights into the modular architecture of PER2:BRCA1 interactions, they represent an initial roadmap for understanding the binding interface. Fragment constructs may not fully recapitulate the binding dynamics, cooperativity, or allosteric regulation present in full-length protein complexes. Nevertheless, the convergence of our fragment-based biochemical data with full-length protein co-immunoprecipitation studies (Fig. 1C and D), structural predictions showing consistent positioning of high-confidence regions in fragment and full-length models (Fig. 2D), and functional validation through ChIP assays demonstrating promoter occupancy by full-length proteins (Fig. 3D) collectively support the biological relevance of these interactions.

### 3.3 Structural modeling supports multiple PER2:BRCA1 binding interfaces

To gain structural insights into PER2:BRCA1 interactions, we performed computational protein-protein interface predictions using AlphaPulldown 2.0. We modeled both a full-length PER2:BRCA1 complex and a fragment complex comprising PER2 (356–574) with BRCA1 (1670–1863) BRCT domains (Fig. 2D). Model confidence was assessed using per-residue predicted local distance difference test (pLDDT) scores, with regions scoring ≥60 considered high confidence, residues 350 to 475 in PER2, and suitable for structural interpretation. Only these high-confidence regions were used to infer potential binding interfaces and to perform contact analysis as shown in Fig. 2D. Regions with pLDDT <60 were classified as low confidence and were not used to draw structural conclusions, though they were retained in models for completeness.

Comparison of the full-length and fragment PER2-BRCA1 complexes revealed consistent positioning of high-confidence regions. Notably, the BRCA1 BRCT region (residues 1670–1863) and a “core” region of PER2 (residues 350–475) exhibited similar positioning, structure, and spatial proximity in both models, providing computational support for the direct interaction between these domains observed in our pull-down experiments. Within the full-length complex, an extended BRCA1 BRCT region (residues 1655–1839) formed multiple predicted interaction sites with PER2 (pLDDT≥60, Fig. 2D).

Quantitative analysis of predicted interaction interfaces revealed that the PER2-BRCA1 complex forms substantial atomic contacts (361 contacts), suggesting an extensive binding interface between PER2 and BRCA1. While the relatively low pLDDT scores in certain regions preclude definitive structural claims, the predicted high-confidence interface regions align well with the experimentally validated binding domains identified through pull-down assays, particularly the PER2(356–574) fragment and the BRCA1 BRCT domains. These computational predictions provide a structural framework for understanding how PER2 engages BRCA1 through multiple discrete interfaces involving both the N-terminal region and C-terminal BRCT domains of BRCA1. Overall, while fragment-based studies provide essential mechanistic insights into the modular architecture of PER2:BRCA1 interactions, they represent an initial approach for understanding binding interfaces. While fragment constructs may not fully recapitulate the binding dynamics or cooperativity of full-length protein complexes, the convergence of our fragment-based biochemical data (Fig. 2A and B) with full-length protein co-immunoprecipitation studies (Fig. 1C and D), and consistent positioning of high-confidence regions in structural models (Fig. 2D) validate these interactions.

### 3.4 PER2, BRCA1, and POU2F1(OCT-1) converge at the promoter of *ESR1*

Having established direct physical interactions between PER2 and BRCA1, we next investigated whether these proteins function together in a relevant transcriptional regulatory context. We focused on the *ESR1* gene, which encodes ERα, a critical determinant of mammary gland development and breast cancer biology (Porras et al., 2021; Lung et al., 2020). *ESR1* transcription is driven primarily by two tissue-specific promoters, A and B (referred to as proAB hereafter), which direct ERα expression in breast epithelial cells (Lung et al., 2020). Previous studies have implicated BRCA1 in transcriptional regulation at the *ESR1* promoter: chromatin immunoprecipitation experiments demonstrated that POU2F1(OCT-1) recruits BRCA1 to proAB, and BRCA1 silencing reduced proAB-driven reporter activity by approximately 50 % (Hosey et al., 2007) Additional cis-regulatory elements critical for *ESR1* transcription have been identified within this region, including two Sp1 family binding sites, a non-canonical E-box element (deGraffenried et al., 2004), and two GATA-3 binding sites that mediate estradiol-dependent transcriptional responses in hormone-responsive breast cancer (Eeckhoute et al., 2007).

To determine whether PER2 might participate in *ESR1* transcriptional regulation alongside BRCA1, we first analyzed the proAB regulatory region (−255 to +144 bp) for putative transcription factor binding sites. Using JASPAR motif analysis (Rauluseviciute et al., 2024), we identified a high-confidence match to the POU2F1(OCT-1) consensus binding motif (5′-ATGCA (A/T/G)AT-3′) located at position −56 to −42 bp (Fig. 3A). This octamer motif is positioned in close proximity to the previously characterized Sp1 binding sites (at −228 and + 77 bp) and overlaps with the non-canonical E-box element (5′CANNTG3′) identified by deGraffenried et al. (deGraffenried et al., 2004). The spatial arrangement of these elements, POU2F1(OCT-1) binding site, Sp1 sites, and the non-canonical E-box, within a compact ∼200 bp region suggests the formation of a higher-order transcriptional regulatory complex. The presence of a POU2F1(OCT-1) binding site may provide a molecular explanation for the BRCA1:POU2F1(OCT-1) interaction at the *ESR1* promoter, as BRCA1 has been shown to function as a transcriptional co-regulator by associating with sequence-specific DNA-binding proteins rather than binding DNA directly (Nadeau et al., 2000). Together, these observations establish the *ESR1* proAB region as a potential convergence point for PER2, BRCA1, and POU2F1(OCT-1), wherein POU may serve as the DNA-binding platform that recruits both BRCA1 and PER2 to coordinately regulate ERα expression.

To functionally validate the predicted POU2F1(OCT-1) binding site to the *ESR1* promoter, we carried out chromatin immunoprecipitation (ChIP) assays in MCF-7 cells transfected with the myc-tagged DNA binding domain (POU) of POU2F1(OCT-1) or empty vector control. ChIP-PCR analysis using primers flanking the predicted binding site demonstrated robust enrichment of the *ESR1* promoter in FLAG-POU immunoprecipitates compared to empty vector (EV) (Fig. 3B, upper panel). RNA polymerase II (RNAPII) served as a positive control, showing expected enrichment at the *ESR1* transcriptional start site. To assess the functional consequence of POU binding, we performed luciferase reporter assays using a construct containing the *ESR1* promoter region cloned upstream of the firefly luciferase gene (pGL2-*ESR1*). Co-transfection of increasing amounts of (myc)_6_-POU expression vector (50–200 ng) with pGL2-*ESR1* reporter resulted in a dose-dependent increase in luciferase activity (Fig. 3B, lower panel). These results demonstrate that POU directly binds to the predicted response element and activates transcription from the *ESR1* promoter in breast cancer cells.

Previous studies have demonstrated that PER2 is essential for assembly of a repressor complex at POU2F1(OCT-1) response elements on promoters of genes involved in epithelial-mesenchymal transition, with both proteins co-immunoprecipitating in breast cancer cells (Hwang-Verslues et al., 2013). Given BRCA1's established role in breast cancer biology and our findings that PER2 associates with BRCA1, we sought to determine whether PER2 directly interacts with the POU DNA-binding domain or requires adaptor proteins such as BRCA1 for recruitment. First, we showed that (myc)_6_-POU co-immunoprecipitates endogenous Per2 in CHO cells (Fig. 1C, lane 3). Furthermore, co-immunoprecipitation in transfected MCF-7 breast cancer cells demonstrated that (myc)_6_-PER2 associated with FLAG-POU (Fig. 1D, lane 3), confirming the interaction is conserved across cell types, suggesting it represents a general transcriptional regulatory mechanism rather than a breast-specific phenomenon.

Next, we asked whether BRCA1 might play a role in PER2:POU interaction. To determine whether PER2, POU, and BRCA1 can assemble into a ternary complex, we performed sequential pull-down assays using recombinant proteins. FLAG-POU DNA binding domain immobilized on anti-FLAG agarose beads was first incubated with *in vitro* translated, [^35^S]-labeled (myc)_6_-BRCA1 (1–400) or (myc)_6_-BRCA1 (1670–1863) to allow binary complex formation. Following extensive washing to remove unbound proteins, beads were re-incubated with [^35^S]-labeled (myc)_6_-PER2 (Fig. 3C). As expected from previous results (Fig. 1C–D), recombinant FLAG-POU bound independently to (myc)_6_-PER2 (lane 4), (myc)_6_-BRCA1 (1–400) (lane 5), and (myc)_6_-BRCA1 (1670–1863) (lane 6). Importantly, when FLAG-POU was pre-incubated with either BRCA1 fragment followed by addition of (myc)_6_-PER2, both BRCA1 and PER2 were retained on the complex (lanes 7 and 8), demonstrating simultaneous binding. These results establish that POU, BRCA1, and PER2 can form a stable ternary complex *in vitro*, wherein POU simultaneously engages both BRCA1 and PER2, providing a molecular framework for their coordinated recruitment to POU2F1(OCT-1) binding sites at target gene promoters.

We then asked whether PER2 and BRCA1 bind to the same POU2F1(OCT-1) response element in *ESR1* and whether their binding absolutely requires prior association of, at least, the POU binding domain of POU2F1(OCT-1) to DNA. Accordingly, we transfected HEK-293T cells with either (myc)_6_-POU, -PER2, -BRCA1 alone or in combination with FLAG-POU as indicated in Fig. 3D and carried chromatin immunoprecipitation studies. We chose HEK-293T cells because of their lack of complex tissue-specific regulatory programs that facilitates dissecting direct transcriptional regulatory mechanisms. In addition, ChIP amplifications were carried out around the flanking region of the POU2F1(OCT-1) response element (−56 to −42) and excluded the Sp1 sites. Our ChIP results show that whereas the POU DNA binding domain is sufficient to recognize and bind the POU2F1(OCT-1) element in the proAB site of *ESR1* (Fig. 3D, lane 6), neither PER2 nor BRCA1 alone was directly able to do it (Fig. 3D, lanes 15 and 18). Conversely, both PER2 and BRCA1 bind to the POU2F1(OCT-1) response element when co-transfected with FLAG-POU suggesting that binding of the later to DNA helps recruiting BRCA1 and PER2 to the complex (Fig. 3D, lanes 9, and 12).

To gain structural insights into the PER2:POU2F1(OCT-1) interaction, we performed computational interface analysis using AlphaPulldown-predicted complex structures. Analysis of the predicted PER2:POU2F1(OCT-1) complex focused on high-confidence regions at the PER2:POU2F1(OCT-1) domain interface, which were consistent with our *in vitro* experimental observations. Analysis of high-confidence regions at the PER2:POU2F1(OCT-1) interface identified 74 residue-residue contacts distributed across the surface of the POU DNA-binding domain (Fig. 3E). Several polar and electrostatic interaction hotspots clustered near residues R299, Q306, and E330 of POU2F1, which are located within or adjacent to the DNA recognition helix. This spatial arrangement is consistent with a direct PER2:POU2F1(OCT-1) interface rather than indirect association through distal structural elements. Notably, the PER2:POU2F1(OCT-1) complex exhibited substantially fewer predicted contacts (74) compared to the PER2:BRCA1 complex (361 contacts), suggesting that PER2 engages POU2F1(OCT-1) through a more limited binding interface that may facilitate dynamic assembly and disassembly of the transcriptional regulatory complex (Fig. 3F). Although individual contacts cannot be experimentally validated at this resolution, the predicted interface regions are consistent with our *in vitro* co-immunoprecipitation results (Fig. 1C and D), supporting the biological relevance of the PER2:POU interaction and its potential role in transcriptional regulation at POU2F1(OCT-1) binding sites.

### 3.5 Mapping the POU2F1(OCT-1) DNA-binding interface reveals ordered recruitment of PER2

**Fig. 4:**
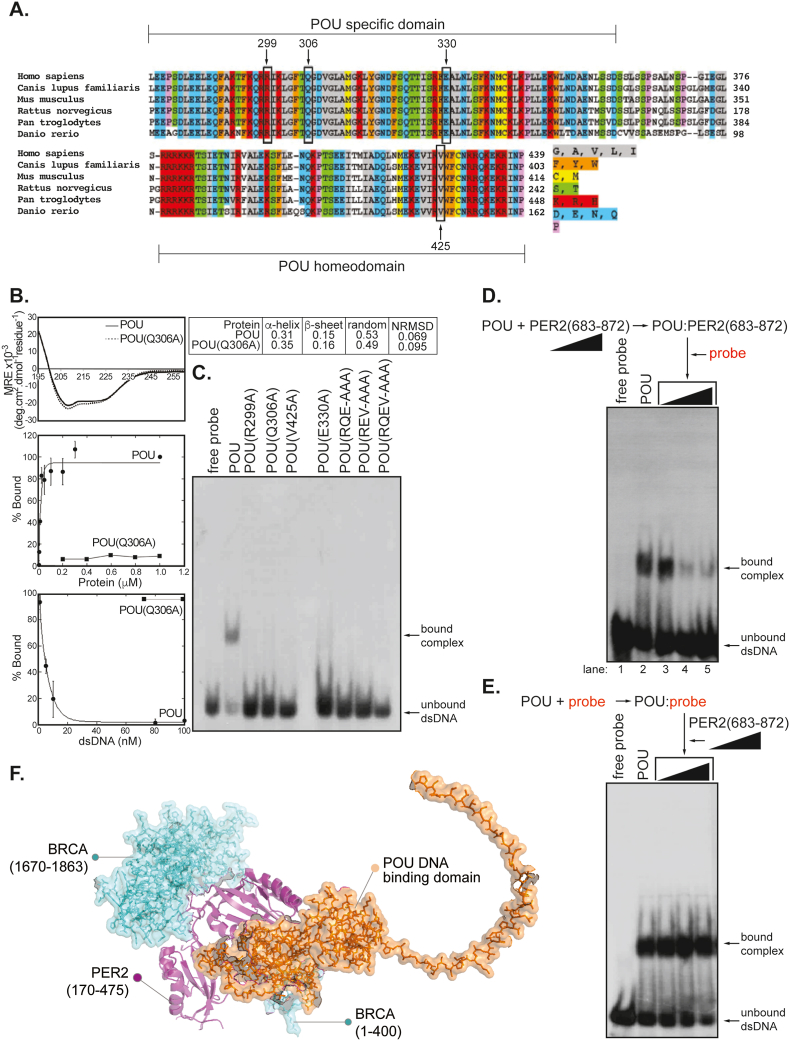
**PER2:POU2 DNA-binding domain interaction reveals multi-component scaffolding with ordered assembly.** (**A**) Sequence alignment of the POU DNA-binding domain across vertebrate species, highlighting highly conserved residues (R299, Q306, E330, and V425) within the POU-specific domain and homeodomain. These residues were targeted for mutagenesis to confirm their contribution to DNA recognition. (**B**) Far-UV circular dichroism (CD) spectroscopy showing that POU point mutations, for example Q306A, do not alter protein secondary structure. Secondary structure content was estimated using DichroWeb with goodness-of-fit validation (NRMSD <0.1) (table). Both wild-type and mutant POU maintain approximately 31–35 % α-helical content and similar β-sheet and random coil proportions, confirming that observed DNA-binding defects result from impaired interaction rather than protein misfolding. (**C**) EMSA showing DNA-binding specificity of wild-type and mutant POU proteins. Radiolabeled double-stranded DNA probe encompassing the POU response element within the *ESR1* promoter (−56 to −42 bp) was incubated with 100 ng purified recombinant wild-type POU or point-mutant POU proteins. DNA-protein complexes were resolved on non-denaturing polyacrylamide gels and visualized by autoradiography. Point mutations at critical interface residues (particularly Q306A and the triple mutant RQE-AAA) severely impair or abolish DNA binding, confirming their essential roles in promoter recognition. (**D**) EMSA demonstrating the effect of pre-assembled PER2:POU DNA binding-domain complexes on DNA binding. Purified recombinant POU was pre-incubated with increasing amounts of PER2(683–872) fragment (up to 5-fold molar excess) before addition of radiolabeled *ESR1* probe. Resulting DNA-protein complexes were resolved on non-denaturing polyacrylamide gels and visualized by autoradiography. Progressive increase in PER2 concentration results in dose-dependent decrease in POU:DNA complex intensity, indicating that pre-assembly of PER2 with POU domain inhibits subsequent DNA binding. (**E**) EMSA showing that DNA:bound POU cannot be displaced by PER2. Purified recombinant POU DNA-binding domain was first incubated with radiolabeled *ESR1* probe to establish a stable POU:DNA complex, followed by addition of increasing amounts of PER2(683–872) (up to 5-fold molar excess). Resulting complexes were resolved and visualized. PER2 fails to disrupt the pre-formed POU:DNA complex or generate a super-shifted ternary species, even at the highest concentrations, demonstrating the high affinity of the POU:DNA interaction and the inability of PER2 alone to form a stable chromatin-associated complex. (**F**) Structural overlay of independently modeled full-length PER2:BRCA1 (PER2 in purple, BRCA1 in teal) and PER2:POU DNA binding-domain (POU in orange) complexes generated using AlphaPulldown 2.0. The analysis reveals that POU and BRCA1 occupy distinct, spatially separated binding surfaces on PER2, with sufficient steric space to accommodate simultaneous binding of all three proteins. This complementary interaction geometry demonstrates that PER2 functions as a dual-engagement platform capable of simultaneously bridging POU2F1(OCT-1) and BRCA1 at chromatin.

Our ChIP experiments support a model in which PER2 is recruited to the *ESR1* promoter through association with POU2F1(OCT-1). However, because the predicted PER2:POU2F1(OCT-1) interaction interface involves the POU DNA-binding domain (Fig. 3E), a critical mechanistic question arises: does PER2 associate with DNA-bound POU2F1(OCT-1), or does a PER2:POU2F1(OCT-1) complex pre-assemble prior to DNA engagement? To distinguish between these recruitment mechanisms, we first mapped conserved residues within the POU DNA-binding domain that are critical for DNA recognition, based on the previously reported structure [PDB: 1OCT (Klemm et al., 1994)]. Multiple sequence alignment of human POU specific domain with vertebrate orthologs, combined with structural modeling of POU domain:DNA complexes, revealed highly conserved residues within the POU homeodomain recognition helix and POU-specific subdomain, including R299, Q306, E330, and V425 [Fig. 4A and (Herr et al., 1988)]. These residues are positioned at the protein-DNA interface, and their evolutionary conservation underscores their functional importance for DNA binding.

To validate the functional role of these residues, we performed electrophoretic mobility shift assays (EMSAs) using recombinant wild-type POU DNA-binding domain and a radiolabeled oligonucleotide probe corresponding to the POU2F1(OCT-1) response element at positions −56 to −42 in the *ESR1* promoter. Far-UV circular dichroism spectroscopy confirmed that point mutations introduced at predicted DNA-contacting residues (R299A, Q306A, E330A, V425A, and combination mutants) did not alter the overall secondary structure or folding of the POU domain (Fig. 4B, wild-type and Q306A are shown in upper panel), with all variants maintaining ∼31–35 % α-helical content and NRMSD values < 0.10. Wild-type POU bound the *ESR1* probe with high affinity, exhibiting dose-dependent complex formation with saturation at ∼0.8 μM protein (Fig. 4B, middle panel) and maintaining stable binding across a range of DNA concentrations (Fig. 4B, lower). In contrast, point mutations at critical interface residues, particularly Q306A and the triple mutant RQE-AAA, severely impaired or abolished DNA binding (Fig. 4C), confirming their essential roles in POU-DNA recognition.

Having established conditions for stable POU domain:DNA complex formation, we next investigated the order of assembly by testing whether a fragment of PER2 that associates with the POU DNA-binding domain before or after DNA binding. In the first scenario, the recombinant POU DNA-binding domain was pre-incubated with the PER2(683–872) prior to addition of radiolabeled *ESR1* (−56 to −42) probe. Under these conditions, we observed formation of a protein-DNA complex with mobility identical to POU alone (Fig. 4D, lanes 2–3). However, when increasing amounts of PER2(683–872) were pre-incubated with purified POU domain (up to 5-fold molar excess), the intensity of the POU:DNA complex progressively decreased (Fig. 4D, lanes 3–5), suggesting that pre-assembly of PER2(683–872) with POU interferes with subsequent DNA binding. This competitive inhibition indicates that PER2 binding to the POU DNA-binding domain prevents or destabilizes DNA recognition when the PER2:POU2F1 complex forms prior to DNA engagement.

In the reciprocal experiment, recombinant POU domain was first incubated with the *ESR1* (−56 to −42) probe to establish a stable POU:DNA complex, followed by addition of increasing molar concentrations of purified PER2(683–872) (also up to 5-fold molar excess over purified POU). Under these conditions, PER2(683–872) failed to disrupt the pre-formed POU:DNA complex or generate a super-shifted ternary species (Fig. 4E), even at the highest PER2(683–872) concentrations tested. This demonstrates two critical features of the interaction: first, once POU engages DNA, the high-affinity POU:DNA interface cannot be competed off by PER2(683–872); and second, PER2(683–872) alone cannot form a stable, detectable ternary complex with DNA-bound POU.

The contrasting results from these two experimental configurations, inhibition of DNA binding when PER2(683–872) pre-associates with POU, but inability to disrupt or join pre-formed POU:DNA complexes, suggest that PER2(683–872) binding and DNA binding are mutually exclusive. Our structural predictions (Fig. 3E) support this interpretation, as several predicted PER2:POU contact residues (R299, Q306, E330) are positioned at or near the DNA recognition helix. However, we acknowledge that our current data cannot definitively distinguish between direct competitive overlap, steric hindrance, or allosteric conformational changes as the underlying mechanism. The absence of a super-shifted ternary PER2(683–872):POU-DNA complex in either configuration indicates that additional factors might be required to stabilize PER2 recruitment to chromatin-associated POU2F1(OCT-1). Given that BRCA1 independently binds both PER2 [Fig. 1C (lanes 1–2), D (lanes 4–5); Fig. 2A and B] and POU domain [Fig. 1D (lanes 1–2)], and that all three proteins form a stable ternary complex *in vitro* (Fig. 3C), we propose that BRCA1 functions as a molecular scaffold that facilitates PER2 association with DNA-bound POU2F1(OCT-1) at the *ESR1* promoter, potentially by bridging PER2 to the complex or by inducing conformational changes that accommodate simultaneous PER2 and DNA binding (Fig. 3F). It is important to note that these experiments employed only the PER2(683–872) fragment; full-length PER2, which contains additional protein interaction domains, may exhibit different binding dynamics in the context of the complete transcriptional complex.

Overall, these results indicate that PER2 association with POU prior to DNA binding inhibits promoter recognition, suggesting that productive recruitment of PER2 to the *ESR1* promoter likely occurs after POU2F1(OCT-1) engages DNA, with BRCA1 playing a critical role in stabilizing this chromatin-associated complex. To understand how all three proteins can simultaneously occupy the *ESR1* promoter complex, we performed structural alignment of the independently modeled full-length PER2:BRCA1 and PER2:POU complexes. This analysis revealed that POU and BRCA1 occupy distinct, spatially separated binding surfaces on PER2 (Fig. 4F), with sufficient steric space to accommodate simultaneous binding of all three proteins within a single complex. While these predictions are based on independently generated models and require experimental validation, the complementary interaction geometry suggests that PER2 can function as a dual-engagement platform, simultaneously bridging POU2F1(OCT-1) and BRCA1 at chromatin. This spatial arrangement supports a model in which BRCA1 facilitates PER2 recruitment to DNA-bound POU2F1(OCT-1) by leveraging distinct PER2 interaction surfaces, enabling formation of a functional tripartite regulatory complex. The ordered assembly mechanism, DNA binding by POU2F1(OCT-1) first, followed by BRCA1-mediated recruitment of PER2, may ensure that PER2-dependent transcriptional regulation is temporally coordinated with POU2F1(OCT-1) occupancy at target promoters, providing a molecular basis for circadian gating of ERα expression.

### 3.6 PER2 imposes circadian gating on BRCA1:POU2F1(OCT-1)-mediated *Esr1* regulation

**Fig. 5:**
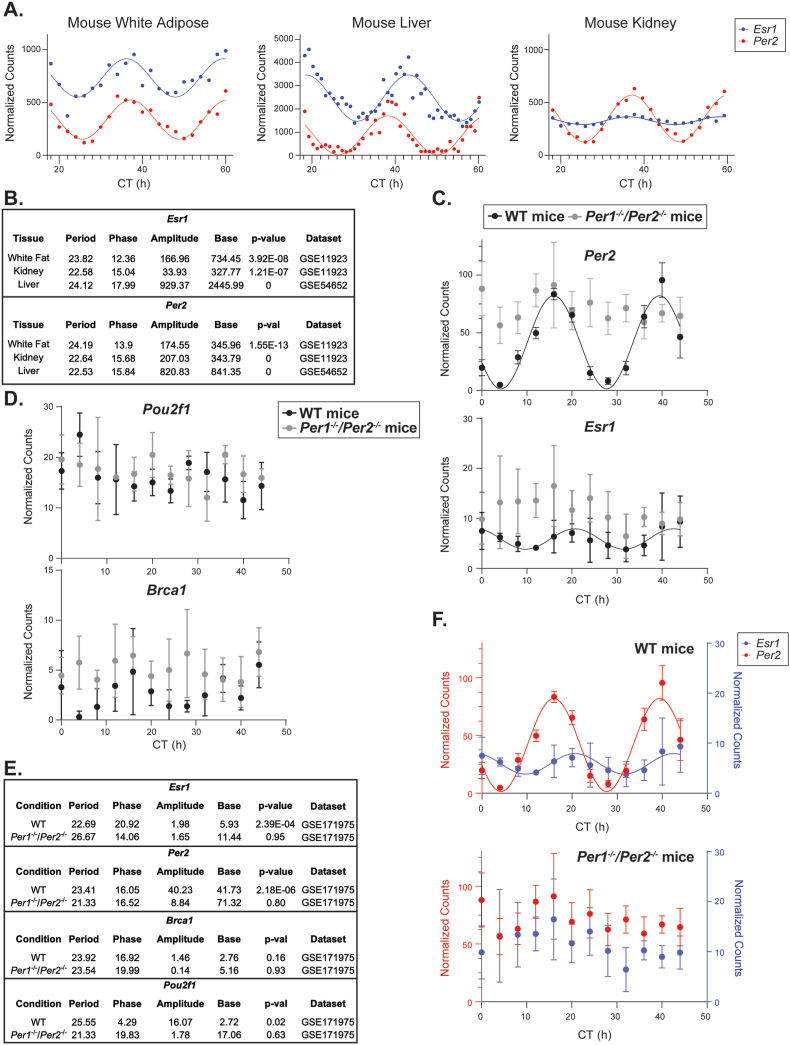
**PER2 integrates circadian and transcriptional signals at the mouse *Esr1* promoter.** (**A**) Time-course expression profiles of *Esr1* and *Per2* mRNA showing circadian oscillations in multiple mouse tissues. Wild-type mice were entrained to 12-h light:12-h dark cycles for one week, then released into constant darkness. Tissues (white adipose tissue, liver, and kidney) were collected at 2 h intervals for 48 h mRNA expression levels (normalized counts) are plotted against circadian time (CT). Fitted cosine curves (blue for *Esr1*, red for *Per2*) show that both genes oscillate with similar periodicity across tissues, with tissue-specific phase relationships. (**B**) Quantitative circadian parameters (period, phase, amplitude, and baseline expression) determined by meta2d analysis of data shown in panel A. Upper table. Circadian periods for *Esr1* and *Per2* in each tissue show remarkable similarity (white adipose: ∼24.2 h for both; liver: ∼23.8 h *Esr1*, ∼23.5 h *Per2*; kidney: ∼24.1 h for both). Lower table. Phase analysis reveals tissue-specific phase relationships: *Esr1* leads *Per2* by 2–4 h in white adipose tissue and kidney, while lagging by ∼1–2 h in liver. P-values (p < 0.01) confirm statistically significant rhythmicity for both genes in all tissues. (**C**) *Per2* and *Esr1* circadian rhythms are abolished in *Per1*^−/−^/*Per2*^−/−^ double knockout mice. Liver expression data from wild-type and *Per1/2* dKO mice (GSE171975) show that in wild-type liver, both *Per2* and *Esr1* exhibit robust circadian oscillations (period ∼23 h, p < 0.01). In contrast, both genes completely lose rhythmicity in *Per1/2* dKO liver tissue, with expression remaining flat across all time points. Importantly, basal transcript levels are maintained despite loss of oscillation, demonstrating that *Esr1* transcription per se does not require functional PER proteins, but rather that circadian modulation of expression requires clock function. (**D**) *Pou2f1* and *Brca1* transcripts show no circadian rhythmicity at the mRNA level. Upper panel: *Pou2f1* expression remains relatively constant across the circadian cycle in both wild-type and *Per1/2* dKO liver, with no statistically significant oscillations. Lower panel: *Brca1* expression similarly lacks circadian rhythmicity in both genotypes. These non-rhythmic expression patterns contrast sharply with the robust oscillations observed for *Per2* and *Esr1*, suggesting that constitutively expressed POU2F1(OCT-1) and BRCA1 serve as stable scaffolding factors that cooperate with rhythmically expressed PER2 to impose circadian control on *Esr1* transcription. (**E**) Meta2d analysis results for *Brca1* and *Pou2f1* showing circadian parameters and statistical assessment. p-values confirm the absence of significant circadian rhythmicity (p > 0.01) for both genes in both wild-type and *Per1/2* dKO animals. (**F**) Direct temporal comparison of *Per2* and *Esr1* oscillations in wild-type and *Per1/2* dKO liver. *Per2* (red) and *Esr1* (blue) expression from GSE171975 are plotted together to visualize their temporal relationship.

To investigate whether *Esr1* expression is regulated in a circadian manner and whether this regulation is coordinated with PER2, we analyzed publicly available time-course transcriptomic datasets from multiple mouse tissues (Hughes et al., 2009; Zhang et al., 2014b). Analysis of wild-type mice maintained under constant darkness revealed that *Esr1* mRNA oscillated with circadian periodicity in white adipose, liver, and kidney tissues (Fig. 5A). Meta2d algorithm analysis confirmed statistically significant rhythmicity for *Esr1* in all three tissues (P < 0.01). Comparison of circadian parameters between *Esr1* and *Per2* revealed that both genes exhibited similar periods across tissues: white adipose tissue (∼24.2 h for both), liver (∼23.8 h for *Esr1*, ∼23.5 h for *Per2*), and kidney (∼24.1 h for both) (Fig. 5B, upper table). Phase analysis showed that *Esr1* and *Per2* oscillations were phase-offset across tissues, with *Esr1* leading *Per2* by approximately 2–4 h in white adipose tissue and kidney but lagging by ∼1–2 h in liver (Fig. 5B, lower table). These tissue-specific phase relationships likely reflect distinct biological roles of ERα in each tissue: in metabolically active adipose tissue and liver, early *Esr1* expression (phase advance relative to *Per2*) may prime estrogen-responsive metabolic gene programs; in kidney, the phase lag may facilitate coupling of circadian hormonal signaling with filtration and reabsorption cycles.

To determine whether circadian *Esr1* expression requires a functional circadian clock, we analyzed liver transcriptome data from *Per1*^−/−^/*Per2*^−/−^ double knockout (dKO) mice (Aviram et al., 2021). In wild-type liver, both *Per2* and *Esr1* exhibited robust circadian oscillations with a period of approximately 23 h (Fig. 5C, black lines), with phase offset similar to that observed in the wild-type tissue analysis (Hughes et al., 2009). In striking contrast, *Per2* and *Esr1* completely lost circadian rhythmicity in liver tissue from dKO mice (Fig. 5C, grey lines), despite maintaining basal transcript levels. The persistent basal expression of *Esr1* in the absence of circadian oscillation demonstrates that *Esr1* transcription per se does not require functional PER proteins, but rather that the circadian modulation of *Esr1* expression is clock-dependent. This finding definitively establishes that *Esr1* circadian rhythmicity requires a functional molecular clock.

We next investigated whether POU2F1(OCT-1) and BRCA1, the transcriptional co-regulators identified in this study, also exhibit circadian expression patterns that might coordinate with *Esr1* regulation. Circadian rhythm analysis of *Pou2f1* and *Brca1* in wild-type mouse liver revealed no statistically significant rhythmicity for both genes in either wild-type or dKO mice (P > 0.01) (Fig. 5D and E). Both *Pou2f1* and *Brca1* maintained relatively constant expression levels across the circadian cycle in both genotypes. This finding supports a model in which constitutively expressed POU2F1(OCT-1) and BRCA1 proteins serve as stable scaffolding factors that cooperate with rhythmically expressed or rhythmically recruited PER2 to impose circadian regulation on *Esr1* transcription. The absence of circadian oscillation for *Pou2f1* and *Brca1* mRNA, combined with their essential roles in *Esr1* transcriptional control (demonstrated in our promoter analysis), indicates that PER2 likely serves as the rate-limiting circadian regulator within this complex, with its oscillating levels and/or nuclear availability dictating the circadian pattern of *Esr1* expression. Lastly, *Per2* and *Esr1* genes show a slight phase-offset oscillations in wild-type animals; however, both genes lose all rhythmicity in *2* dKO liver, establishing that functional PER1 and PER2 are required for maintaining *Esr1* circadian expression (Fig. 5F).

**Fig. 6:**
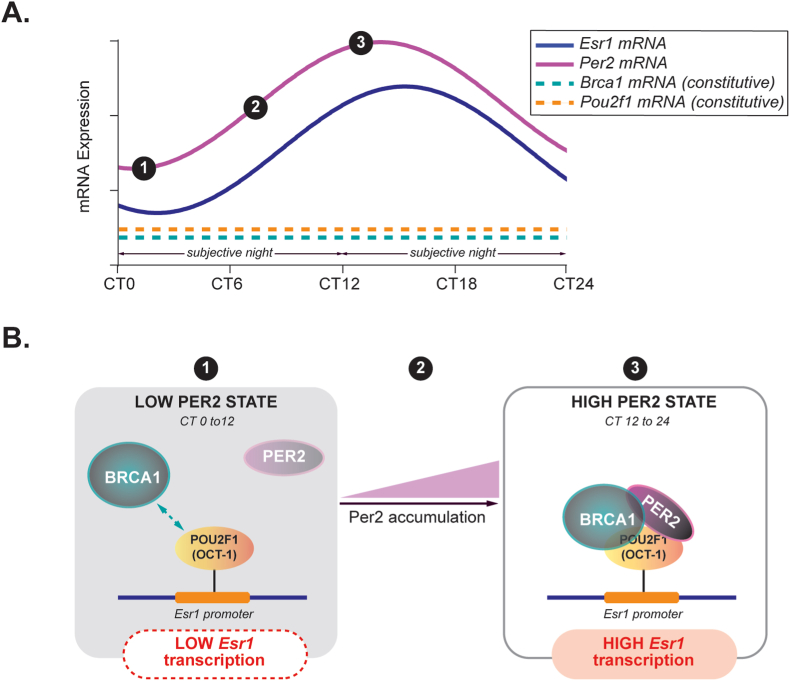
**Model of PER2-Mediated Circadian Gating of *Esr1* Transcription Through Ordered Ternary Complex Assembly.** (**A**) Circadian expression dynamics across the 24-h cycle. Time-course analysis of mRNA expression patterns shows that *Per2* (purple line) and *Esr1* (blue line) exhibit robust circadian oscillations with peak expression during the early phase of the subjective day (CT 12–18) and trough expression during the subjective night (CT 0–6). *Per2* and *Esr1* oscillate in phase with maximal expression around CT 8–12. In contrast, *Brca1* (teal dashed line) and *Pou2f1* (orange dashed line) mRNAs are constitutively expressed throughout the circadian cycle without significant rhythmicity. Data are based on circadian transcriptomics analyses in wild-type mouse tissues (see Fig. 5). CT, Circadian Time. (**B**) Molecular mechanism of circadian-gated transcriptional regulation. The model depicts two temporal states separated by circadian time progression (black arrow). Left panel (LOW PER2 STATE): During the resting phase and the beginning of the active phase (CT 18-6), when PER2 protein levels are low, POU2F1(OCT-1) (orange oval) is constitutively bound to its response element (orange box) within the *Esr1* promoter (blue bar). BRCA1 proteins (teal oval) is present in the nucleus but inefficiently recruited to the promoter. Sparse PER2 molecules (small faded purple ovals) fail to nucleate stable ternary complex assembly. Under these conditions, *Esr1* transcription remains low (red dashed box). Right panel (HIGH PER2 STATE): During the end of subjective night (CT 6–12), when PER2 protein accumulates to high levels, abundant PER2 (large purple oval) engages both BRCA1 domains simultaneously through multivalent binding interactions. BRCA1 serves as a molecular bridge connecting PER2 to DNA-bound POU2F1(OCT-1). This ordered assembly, requiring POU2F1(OCT-1) to bind DNA first, followed by BRCA1 scaffolding and PER2 recruitment, forms a functional ternary complex at the *Esr1* promoter. The assembled complex drives high *Esr1* transcription (red filled box). Numbered boxes at top indicate key features: (1) Ordered Assembly: Complex formation follows a specific sequence (POU2F1 → DNA → BRCA1 → PER2). Pre-assembly of PER2 with POU2F1(OCT-1) before DNA binding inhibits complex formation (see Fig. 4). (2) Circadian Gating: PER2 protein levels oscillate with circadian rhythms, providing temporal control of *Esr1* transcription. BRCA1 and POU2F1(OCT-1) are constitutively expressed, functioning as stable platform components that are temporally gated by rhythmic PER2 availability. (3) Multivalent Binding: PER2 engages BRCA1 through two discrete interfaces (PER2 residues 356–574 and 683–872 bind BRCA1 residues 1–400 and 1670–1863, respectively), ensuring stable ternary complex formation and robust transcriptional activation.

In summary, these circadian expression analyses establish *Esr1* as a bona fide clock-controlled gene whose rhythmic expression requires functional PER proteins, while specific roles established in the PER2:BRCA1:POU2F1(OCT-1) regulatory complex: PER2 functions as the circadian timing component whose oscillations gate transcription, while BRCA1 and POU2F1(OCT-1) provide constitutive scaffolding and DNA-binding functions necessary for promoter recognition and transcriptional control (Fig. 6).

## 4 Discussion

The architectural principles of the PER2:BRCA1:POU2F1(OCT-1) complex reveal an elegant regulatory strategy wherein constitutively expressed DNA-binding [POU2F1(OCT-1)] and scaffolding (BRCA1) components provide stable platforms that are temporally gated by rhythmically oscillating PER2. This design principle, stable scaffold plus rhythmic regulator, may represent a generalizable mechanism for achieving both specificity and temporal precision in circadian gene regulation. Unlike regulatory models where all components oscillate in synchrony, this architecture allows a single rhythmic component (PER2) to impose circadian control on multiple target genes through combinatorial assembly with different constitutive transcription factors, thereby expanding the regulatory repertoire of the circadian clock without requiring clock-dependent expression of every regulatory component*.* BRCA1 has emerged as a versatile scaffold that assembles with multiple transcription factors and co-regulators to control gene expression. For instance, BRCA1 interacts with STAT1 to differentially regulate interferon-gamma target genes, wherein BRCA1 recruits STAT1 to chromatin and modulates its transcriptional output without directly binding DNA (Ouchi et al., 2000). Similarly, BRCA1 functions as a co-activator of estrogen receptor in the presence of estradiol, enhancing ER-mediated transcription of hormone-responsive genes through protein-protein interactions rather than autonomous DNA binding (Fan et al., 2001). In these contexts, BRCA1 consistently serves as a bridge between sequence-specific DNA-binding proteins and the transcriptional machinery, a role that parallels its function in the PER2-POU2F1(OCT-1) complex.

Other characterized ternary complexes involving POU2F1(OCT-1) share similar architectural principles but have distinct functional outcomes. POU2F1(OCT-1) has been shown to cooperate with transcription factors including SOX2 and SOX4, GATA-3, Bob1, and more generally TFIIB, to regulate housekeeping and tissue-specific genes at promoter regions, where its binding facilitates the recruitment of co-activator complexes (Lee et al., 2001; Donner et al., 2007; Nakshatri et al., 1995; Kim et al., 2016). However, these complexes typically operate through cooperative DNA binding and chromatin remodeling, mechanisms that differ fundamentally from the PER2:BRCA1:POU2F1(OCT-1) architecture. The ordered assembly mechanism we propose, wherein POU2F1(OCT-1) must engage DNA prior to stable PER2 recruitment, is supported by our EMSA studies showing that pre-assembly of PER2 with POU inhibits subsequent DNA binding (Fig. 4D), while DNA-bound POU cannot be displaced by PER2 alone (Fig. 4E). Importantly, our ChIP experiments (Fig. 3D) demonstrate that in intact cells, BRCA1 is essential for stabilizing PER2 at POU-bound chromatin, supporting a model wherein BRCA1 serves as a molecular scaffold that facilitates PER2 association with DNA-engaged POU2F1(OCT-1) at target promoters. In the PER2:BRCA1:POU2F1(OCT-1) complex, neither BRCA1 nor PER2 independently engages the *ESR1* promoter; instead, they are recruited exclusively through their respective interactions with DNA-bound POU2F1(OCT-1). This hierarchical dependency represents a distinct feature that confers greater specificity compared with models in which multiple components independently recognize DNA.

The role of BRCA1 as a generalized scaffolding factor extends beyond transcriptional regulation into DNA repair, cell cycle checkpoint, and chromatin regulation. BRCA1 interacts with numerous proteins including RAD51 (facilitating homologous recombination), CHK2 (in cell cycle checkpoint signaling), and BACH1 (a helicase involved in DNA repair) (for review see (Savage and Harkin, 2015) and references within). These interactions establish BRCA1 as a hub protein that organizes multiprotein complexes performing distinct cellular functions. The dual functionality of BRCA1, serving simultaneously as a tumor suppressor involved in DNA repair and as a circadian transcriptional regulator, suggests that hub proteins like BRCA1 may integrate signals across multiple cellular pathways. Evidence indicates that DNA damage and circadian signaling pathways are functionally coupled, with circadian clocks influencing the timing and efficiency of DNA repair processes (Bee et al., 2015; Gaddameedhi et al., 2011). The PER2-BRCA1 interaction may therefore represent a molecular integration point where circadian timing influences not only hormone signaling but also the coordination of DNA repair with circadian phases.

Rather than a single, universal regulatory mechanism, clock-controlled genes may be regulated through tissue- and gene-specific combinations of clock proteins and co-regulators. This context-dependent regulation could explain the considerable heterogeneity in circadian gene expression patterns observed across tissues and cell types (Zhang et al., 2014a; Hughes et al., 2009). The discovery of BRCA1 as a circadian co-regulator suggests that other canonical tumor suppressors or DNA damage response proteins may similarly participate in circadian transcriptional control at specific target genes.

The hierarchical dependence of this ternary complex on BRCA1 function creates vulnerability to two distinct regulatory disruptions. First, BRCA1 loss-of-function mutations, which are common in hereditary breast cancer, would be predicted to impair BRCA1-mediated stabilization of PER2 recruitment to the *ESR1* promoter. This would disrupt circadian gating of *ESR1* transcription and result in dysregulated ERα expression and aberrant estrogen signaling (Wang et al., 2018). By providing a molecular mechanism that links BRCA1 deficiency directly to circadian disruption of hormone-responsive gene expression, our findings offer a new perspective on BRCA1-associated cancer predisposition beyond its canonical roles in DNA repair. Second, circadian misalignment induced by shift work or chronic jet lag represents an alternative route to complex dysfunction. Circadian disruption reduces both the amplitude and nuclear availability of PER2, compromising its ability to serve as the temporal regulator within the PER2-BRCA1-POU2F1(OCT-1) complex. Epidemiological studies have consistently associated shift work with increased incidence and poorer outcomes in hormone-dependent malignancies including breast, prostate, thyroid, and endometrial cancers (Schernhammer et al., 2003; Malaguarnera et al., 2020; Zhou et al., 2022; Jagielo et al., 2023). Our mechanistic findings suggest that circadian misalignment dysregulates *Esr1* expression through impaired PER2 function, thereby increasing estrogen signaling and cancer risk in shift-workers. The multi-component architecture of this regulatory complex suggests multiple therapeutic interventions: stabilizing PER2 protein levels, enhancing BRCA1-PER2 interaction, modulating BRCA1 scaffolding specificity, or restoring circadian alignment. Understanding how each disruption, BRCA1 mutation or circadian misalignment, impairs complex assembly could inform rational therapeutic strategies for restoring circadian *Esr1* control in hormone-dependent cancers and for mitigating cancer risk in populations exposed to circadian disruption.

While our transcriptomic analyses establish that *Esr1* exhibits robust circadian oscillations that require functional PER proteins, and our biochemical studies demonstrate that PER2, BRCA1, and POU2F1(OCT-1) form a ternary complex that regulates *Esr1* promoter activity, the study does not assess the downstream functional consequences of circadian *Esr1* regulation. Future studies examining whether ERα target genes (such as progesterone receptor, TFF1/pS2, or GREB1) exhibit circadian expression patterns, whether these oscillations are disrupted in Per1/2 knockout animals, and whether circadian misalignment affects estrogen-responsive phenotypes (such as hormonal cycling, mammary gland development, or estrogen-dependent tumor growth) will be essential for establishing the physiological and pathological relevance of circadian *Esr1* control. Such studies would provide critical insights into how circadian disruption may contribute to hormone-dependent disease pathogenesis.

In conclusion, the PER2-BRCA1-POU2F1 ternary complex represents a sophisticated regulatory architecture that integrates multi-component scaffolding with ordered assembly kinetics to achieve temporal control of gene expression (Fig. 6). While BRCA1 has well-characterized roles as a scaffolding factor in DNA repair and checkpoint control, its function in the circadian regulation of *ESR1* reveals previously unappreciated connections between tumor suppression pathways and circadian biology. The hierarchical assembly of this complex, with constitutively expressed DNA-binding and scaffolding components coordinated by a rhythmically oscillating timing factor, may represent a generalizable principle for achieving both specificity and temporal precision in the regulation of clock-controlled genes.

## CRediT authorship contribution statement

**Elizaveta Kadukhina:** Writing – original draft, Investigation, Formal analysis, Data curation. **Siqi Jia:** Writing – original draft, Investigation, Data curation. **Linda M. Villa:** Investigation. **Xiao Yi:** Investigation. **Daniel G.S. Capelluto:** Writing – review & editing, Resources, Investigation, Formal analysis. **Jonathan S. Briganti:** Writing – review & editing, Visualization, Investigation. **Anne M. Brown:** Writing – review & editing, Visualization, Investigation. **Carla V. Finkielstein:** Writing – review & editing, Visualization, Supervision, Resources, Project administration, Funding acquisition, Conceptualization.

## Funding

This study was funded by Fralin Biomedical Research Institute and Avon Foundation to C.V.F. The funders played no role in study design, data collection, analysis and interpretation of data, or the writing of this manuscript.

## Declaration of competing interest

The authors declare the following financial interests/personal relationships which may be considered as potential competing interestsCarla V. Finkielstein reports financial support was provided by Fralin Biomedical Research Institute and Avon Foundation. If there are other authors, they declare that they have no known competing financial interests or personal relationships that could have appeared to influence the work reported in this paper.

## Data Availability

Datasets used in the article are publicly available and properly acknowledged in the article.
